# Access to sexual and reproductive health services for women living with HIV in Latin America and the Caribbean: systematic review of the literature

**DOI:** 10.1002/jia2.25273

**Published:** 2019-04-08

**Authors:** Marcela Gómez‐Suárez, Maeve B Mello, Mónica Alonso Gonzalez, Massimo Ghidinelli, Freddy Pérez

**Affiliations:** ^1^ The National University of Colombia Interfaculty Doctoral Program in Public Health Bogotá Colombia; ^2^ Department of Communicable Diseases and Environmental Determinants of Health HIV, Hepatitis, Tuberculosis, and Sexually Transmitted Infections Unit Pan American Health Organization Washington DC USA

**Keywords:** HIV seropositive, women living with HIV, women's health, women's rights, sexual and reproductive health services, access to health services, family planning, antenatal care, abortion services, violence against women, Latin America, the Caribbean

## Abstract

**Introduction:**

Systematic reviews show that women living with HIV (WLHIV) have high unmet sexual and reproductive health (SRH) needs due to barriers to access sexual and reproductive health services (SRHS). In Latin America and the Caribbean (LAC), as of 2016, there were nearly one million WLHIV, but the existing evidence of their SRH needs comes from a few individual studies. This systematic review provides an overview of these women's needs to help define new and/or adapt existing public health strategies to the local context. This review synthesizes the evidence from the literature on the use of and access to SRHS related to family planning, antenatal care, abortion services and violence against WLHIV in LAC.

**Methods:**

Using a systematic review of mixed studies, a search was performed in MEDLINE, EMBASE, LILACS, INASP, POPLINE, SCOPUS, for studies conducted in LAC, from 2004 to 2017, as well as contact with authors and hand search as needed. Two independent reviewers evaluated the quality of the studies using the Mixed Methods Appraisal Tool; inclusion was conducted according to the PRISMA flow diagram. An exploratory narrative synthesis followed by quantitative synthesis data was undertaken. Group analysis or meta‐analysis was not considered appropriate given the level of heterogeneity of the studies.

**Results:**

A total of 18 studies in 13 LAC countries for a population of 5672 WLHIV were included. Data from individual studies reported unmet family planning needs; higher, but inconsistent use of condom as the sole contraceptive method OR=1.46 [1.26 to 1.69]; lesser use of other non‐permanent contraceptive methods OR=0.26 [0.22 to 0.31]; more unplanned pregnancies OR=1.30 [1.02 to 1.66]; more induced abortions OR=1.96 [1.60 to 2.39]; higher risk of immediate postpartum sterilization; and higher exposure to sexual and institutional violence by WLHIV when compared with women without HIV.

**Conclusions:**

This review presents evidence from LAC about the SRH unmet needs of WLHIV that must be addressed by decreasing institutional and structural barriers, facilitating services and reducing stigma, and discrimination among healthcare providers to improve access to SRHS based on human rights, so women independently of their HIV status can make their own reproductive decisions, free of violence and coercion.

## 1 Introduction

The right to sexual and reproductive health (SRH) is included in the United Nations international covenant on rights, wherein a woman's reproductive option is considered a basic human right 1. The right to sexual and reproductive health services (SRHS) is understood as the possibility of having easy access to and receiving timely and efficient care that meets the needs of women throughout their life course as well as the freedom to make reproductive decisions. However, studies regarding women living with HIV (WLHIV) and their conditions for accessing SRHS show that their SRH needs are largely unmet 2, leading to high rates of unsafe abortions and three times the number of unplanned pregnancies than women not living with HIV (WNLHIV) 3, 4. In 2017, the World Health Organization (WHO) launched the Consolidated Guideline on Sexual and Reproductive Health and Rights of Women Living with HIV to support countries to implement evidence‐based interventions to improve access to SRHS and contribute to the realization of this basic right 5.

As of 2016, an estimated 2.1 million people were living with HIV in Latin America and the Caribbean (LAC), of which nearly half were women, mostly of reproductive age. WLHIV are vulnerable to unfavourable physical, social and economic factors, which operate at multiple levels and are aggravated by gender inequalities that persist in the Region 6, 7. In the LAC, evidence for the barriers that WLHIV face regarding access and use of SRHS are scarce and have been reported primarily from individual studies conducted in Brazil, Argentina and Mexico 8, 9, 10, 11, 12, 13, 14, 15, but a systematic review focusing on LAC has not been performed. This article presents the results of a systematic review of the literature regarding the use and access of SRHS for WLHIV in LAC to provide an overview of the needs of these women and to help define new and/or adapt existing public health strategies to the local context.

## 2 Methods

In accordance with our registered protocol (PROSPERO ID CRD42017073084), a systematic mixed‐methods review was undertaken to synthesize quantitative, qualitative and mixed studies in English, Spanish and Portuguese published from 2004 to 2017 and conducted among WLHIV of childbearing age in LAC. The outcomes include the use of and access to SRHS associated with family planning, including abortion, and antenatal care. Studies including key populations related to HIV were excluded, as were studies that did not report the methodology used, editorials, letters and commentaries.

Literature was retrieved with a search strategy using the following MeSH terms: HIV, AIDS, healthcare delivery, access to health services, reproductive health, contraception, abortion, women's health, maternal health, the Caribbean region, Central America, Mexico, Latin America and South America. The major health‐related databases MEDLINE, EMBASE, LILACS, CABI, INASP, POPLINE and SCOPUS were searched. Contact with authors and a hand search in Latin American journals was also carried out.

Two independent reviewers evaluated titles and abstracts using the STROBE statement tool 16; in case of disagreement, a third reviewer made the inclusion decision. The full text of the selected studies was retrieved, and two reviewers evaluated the quality of the methodology using the Mixed Methods Appraisal Tool (MMAT), an instrument designed and validated for this type of review 17.

To determine the degree of agreement, Cohen's Kappa (K) was calculated for two independent reviewers in ReCal 2 18. The decision on which studies to select was made using the PRISMA flow diagram 19.

**Figure 1:**
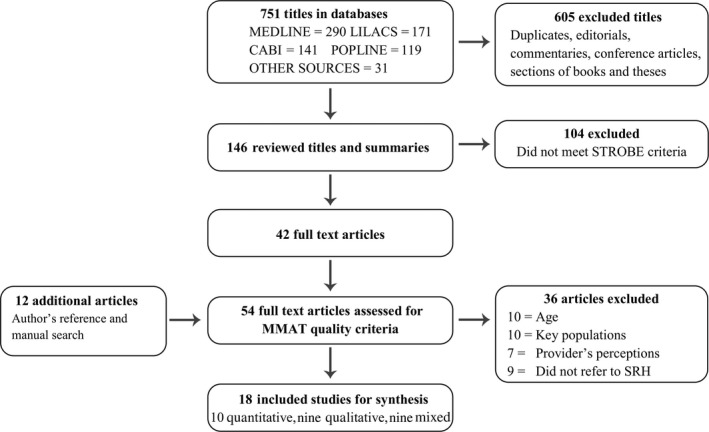
PRISMA flow diagram for identification and conclusion of studies

Initially, 751 titles and abstracts were identified. After exclusions, 146 were selected, of which 42 met STROBE quality criteria and the full text was recovered. Twelve additional studies from direct contact with authors and hand search were recovered for a total of 54 full‐text studies assessed for MMAT quality criteria (K=0.7 quantitative, K=0.8 for qualitative studies, and K=0.6 for mixed studies). The final synthesis included 18 studies: 10 quantitative, four qualitative and four mixed‐methods, in accordance with the PRISMA flow diagram (Figure 1).

An exploratory narrative synthesis was chosen to summarize the results, as described by Pluye 17. Synthesis of qualitative data was conducted using thematic analysis to group the outcomes according to the emerging thematic categories and subcategories and was described in a narrative form. Simultaneously, quantitative data were synthesized by describing measures of frequency and association from individual studies to find differences in the outcomes that may support the qualitative findings and reveal knowledge gaps. Group analysis or meta‐analysis was not considered appropriate given the level of methodological heterogeneity of the studies included.

## 3 Results

A total of 18 studies were included, of which 10 were conducted in South America 8, 9, 10, 11, 12, 13, 15, 20, 21, 22, four in Mesoamerica 14, 23, 24, 25, two in the Caribbean 26, 27 and two multicentric 28, 29, totalling 5,672 WLHIV from 13 LAC countries (Table 1).

**Table 1:** Distribution by country of included studies

Country	No. studies	N	Reference
Mexico	6	460	14, 23, 24, 25, 28, 29
Dominican Republic	1	21	27
El Salvador, Nicaragua, Honduras	1	285	28
Trinidad‐Tobago, Antigua, Barbados	1	230	29
Brazil	7	3062	8, 9, 10, 11, 12, 20, 22
Argentina	3	715	13, 15, 29
Colombia	1	899	21

Of the studies using only quantitative methods, eight included a cross‐sectional design with non‐probabilistic sampling, one was a cohort study and one was a case–control study. The comparison group used in the two‐group studies was WNLHIV. One study used men as controls, but only data from women were included as a one‐group study. In seven studies, authors used stratification and adjustments in the analysis to control for selection bias. Qualitative studies used interpretative phenomenological approaches, while mixed‐methods studies used a sequence of cross‐sectional and interpretative analysis. All studies were conducted in healthcare facilities, with some specialized HIV care and treatment services. The studies are fully described in Appendix 1.

As demographic characteristics of participants were heterogeneously reported in the studies, it was not possible to categorize these variables. Nevertheless, this review showed that WLHIV represents an age group ranging between 25 and 35 years, have an average‐low education (five to seven years), have at least one child, work mainly in the informal sector (medium‐low income) and are not living with a partner. The ethnicity of the participants included black and other Afro‐descendants, mestizo, white and indigenous populations 8, 9, 10, 11, 12, 25, 29.

### 3.1 Qualitative–quantitative synthesis

Four thematic categories emerged and are summarized in Table 2. Results for each are described below.

**Table 2:** Thematic categories/subcategories with quantitative results

	%^a^	I.P^b^	OR^c^ IC 95%	*p*
Family planning				
Contraceptive methods				
Barbosa 8	‐	60.00	1.50 [1.30 to 1.74]	<0.0001
Pilecco 11	‐	56.10	1.28 [1.11 to 1.40]	<0.001
Barbosa 9	‐	59.30	1.46 [1.26 to 1.69]	<0.001
Gogna 13	73.70			
Barcellos 10	62.00			
Dual contraception				
Pilecco 11	8.90			
Barbosa 8	‐	23.00	0.3 [0.22 to 0.41]	
Kendall 14	7.00			
Pregnancy and abortion				
Unplanned pregnancy				
Aguilar 25	‐		8.78 [0.95 to 80.7]	0.23
Pilecco 11	‐	56.00	1.3 [1.02 to 1.66]	
Barcellos 10	65.00			
Kendall 28	67.00			
Gogna 13	55.00			
Voluntary induced abortion				
Pilecco 10	‐	79.70	3.93 [2.06 to 7.47]	
Barbosa 9	‐	66.20	1.96 [1.60 to 2.39]	
Friedman 12	31.00			
Post‐diagnosis sterilization				
Barbosa 8	‐	66.80	2.02 [1.48 to 2.76]	
Pilecco 11	‐	60.30	1.52 [1.29 to 1.79]	
Hopkins 20	‐	82.40	4.7 [2.58 to 8.58]	
Oliveira 22	36.20			
Violence				
Sexual violence				
Aguilar 25	‐	84.10	16.05 [4.35 to 59.8]	<0.0001
Barbosa 8	‐	59.50	1.47 [0.93 to 2.25]	

^a^Percentage reported in one group studies WLHIV; ^b^IP (implied probability) is the value of OR converted to a probability value to facilitate comprehension *p* = OR/1 + OR; ^c^comparissons made within two groups WLHIV versus WNLHIV.

#### 3.1.1 Family planning – use of contraceptives and dual protection

Eight studies investigated the use of non‐permanent contraceptive methods 9, 10, 11, 13, 14, 15, 26, 27. WLHIV reported very low use of non‐permanent contraceptives other than condoms ranging from 6.3% in Argentina to 3.2% in Mexico with pills being the most used, followed by injectable hormonal methods and, lastly, intrauterine device (IUD) 13, 14. In Argentina, emergency contraception was used at some point by 20% of WLHIV, although 80% new about it 15. The qualitative data supported these findings, with WLHIV not using other contraceptive options due to high cost, lack of information and restricted availability in healthcare settings 13, 27. In addition, one study reported misunderstandings by WLHIV that condoms and/or sterilization were the only safe contraceptive options for them at the time 14.

In all the studies, that included this outcome, condom use among WLHIV ranged between 30% in Brazil 10 to 67% in the Caribbean 27. Also, in Brazil, Barbosa et al. referred higher use of condom in WLHIV than WNLHIV at last sexual encounter (44.3% vs. 25.8%) 9. Consistency (during every sex encounter) of condom use, however, was reported to be low in both groups ranging from 30% in Argentina to 60% in the Caribbean 13, 26. A direct association was reported between inconsistent condom use and low education, economic insecurity and sporadic partners. In contrast, serodiscordant stable couples reported a higher consistency of condom use 26. In Argentina, Gogna et al. reported that 60% of WLHIV who had received condoms in their visits to HIV clinics cited difficulties in consistent use due to cultural barriers and limited ability to negotiate condom use with partners 15. Similar findings were observed in Brazil and Mexico 11, 14.

Three quantitative studies in Brazil concluded that WLHIV used condoms as the sole contraceptive method more often than WNLHIV (60% vs. 56% respectively) 8, 9, 11, with similar results found by the two other single‐group studies 10, 13; one conducted in Brazil (62% of condom use) and one in Argentina (74% of condom use).

Qualitative studies identified low use of dual methods among WLHIV, ranging from 15% in the Caribbean 27 to less than 8% in Argentina 13. The probability of using dual methods among WLHIV was 23% lower than in WNLHIV as found in Brazil 8. The qualitative studies encountered emerging difficulties in the offering and availability of contraceptive methods besides condoms in the HIV clinics, additionally to institutional barriers to SRHS such as the absence of counselling on family planning methods to WLHIV in these specialized services 14. Gogna et al. in Argentina identified the fragmentation of healthcare services without clear norms concerning the roles and responsibilities of contraceptive counselling provision to WLHIV 15. Conversely, a study in the Dominican Republic noted that more than half of the HIV services offered dual methods (54% of HIV services) to their clients; however, uptake remained as low as 15%. Reasons attributed to the low use of contraceptives were related to the burden of using oral contraceptives on top of antiretroviral treatment as well as the changes that hormonal contraceptive may have on menstruation in the local cultural beliefs that equate menstrual cycles to women's health and wellbeing 27.

#### 3.1.2 Pregnancy and abortion

Studies from Brazil 10, 11, Mexico 14, 23, 25, Argentina 15 and the Dominican Republic 27 found a higher frequency of unplanned post‐diagnosis pregnancies ranging from 56% to 70%, even though these women expressed intentions of having fewer children than WNLHIV 11. The main factors associated with the occurrence of unplanned post‐diagnosis pregnancies were related to conditions of greater social and economic vulnerability, that is being young (less than 20 years) 25, having suffered violence, having less than five years of education 10, besides having had a miscarriage in previous pregnancies 23, 27. Likewise, 79% more unplanned pregnancies among WLHIV were reported to have ended in induced abortions 11, and this association remained after adjusting for age, parity, education and the number of sexual partners. When asked about the motivations for having an abortion, WLHIV cited fear of perinatal HIV transmission as its main reason in 48% of cases. These results are in agreement with two other Brazilian studies where, after adjusting for age, WLHIV continued having a higher risk of induced abortion than WNLHIV; and 68% of the unplanned pregnancies ended in abortions 9, 12. Fear of transmitting HIV to babies was also cited as the main motivation to seek an abortion.

#### 3.1.3 Post‐diagnosis sterilization

Two studies on post‐diagnosis sterilization were also performed in Brazil using a similar research design, a decade apart. In 2005, Hopkins et al. reported that sterilization was 82% more common among WLHIV than WNLHIV, and it was performed three times more frequently in the immediate postpartum. Most WLHIV who delivered by caesarean section (c‐section) were sterilized, and nearly one in five who delivered vaginally also underwent the procedure 20. Ten years later, Barbosa et al. found no significant difference between WLHIV and WNLHIV regarding the risk of sterilization in the immediate postpartum 8. For both groups of women, high unmet needs for sterilization were reported. However, WLHIV were more likely to be sterilized during a c‐section as compared to WNLHIV. Considering the reported unmet need for sterilization of WLHIV and the existence of national family planning legislation that imposes strong conditions for sterilization during a c‐section, HIV diagnosis was often used as a justification to conduct the procedure, that is WLHIV had their need for voluntary sterilization met more often than WNLHIV. Also, in the Brazilian context, fear of transmitting HIV to the infant was the main reason attributed by WLHIV to undergo voluntary sterilization, which occurred for 96% of mothers in the immediate postpartum, as reported by Oliveira et al. 22.

Interval sterilization was studied in Brazil 8, 22, with the findings highlighting the existence of institutional barriers for WLHIV to access this procedure. The proportion of unmet demand for sterilization was higher among women under the age of 30. In Colombia, Montoya et al. reported no differences in referrals and timing to received care by women with and without HIV in public health services 21.

#### 3.1.4 Violence against WLHIV

In five articles, factors related to sexual, physical and interfamily violence and discrimination against WLHIV were addressed 9, 10, 12, 25, 28. The risk of suffering any kind of violence was reported ranging from 2 to 16 times higher in WLHIV as compared to WNLHIV 9, 25. In a study in Mexico, the disclosure of HIV status to the sexual partner and condom negotiation were the principal triggers for violent acts 25. Similar results were supported by women's narratives captured in qualitative studies conducted in Honduras, El Salvador, Nicaragua 28 and Argentina 13, where sexual, physical and psychological violence were repeatedly mentioned by WLHIV. In articles that addressed institutional violence, WLHIV were shown to experience greater improvement in specialized HIV treatment services with more personalized, friendly care than in the past, but challenges arise when they sought care outside HIV services, mostly related to healthcare providers from maternal‐and‐child health and family planning services 15, 24, 26. In Argentina 15 and Brazil 10, women who were diagnosed with HIV during prenatal care reported more vulnerability to institutional violence because of their lack of knowledge and limited time to internalize the diagnosis. Meanwhile, women who became pregnant after knowing their positive HIV status reported violence due to the stigmatizing assumptions from the healthcare providers that “they were not supposed to get pregnant” 28.

## 4 Discussion

Women's differential vulnerabilities to HIV acquisition have been studied in different contexts and are consistently associated with disadvantageous economic security, education and other structural determinants of health 8, 30, 31, 32. Similarly, such vulnerabilities were also present in the studies conducted in LAC, as the majority of the WLHIV included in this review were young, less‐educated, working in informal jobs and most users of public health services. The thematic categories that emerged from this review showed that, besides the structural health determinants related to HIV, these women also face barriers to access contraceptive alternatives and SRHS. This imposes a limit to their capacity to make autonomous decisions about their SRH based on rights and should be addressed as an important part of the health strategies for these populations 5, 33.

Condoms are an essential component of HIV prevention, but due to the difficulties to negotiate their consistent use between WLHIV and their partners; sole use is not recommended to avoid unplanned pregnancies and should not be the only option for WLHIV 5. However, WLHIV are more likely to use condoms as a sole contraceptive method than WNLHIV, as described in LAC studies and corroborated by a systematic review involving 27 countries worldwide 34, 35 and a recent study performed in Mozambique 36. It is important to note that dual contraception use in LAC by WLHIV was lower than that reported in studies conducted in sub‐Saharan Africa 3.

Furthermore, low use of non‐permanent contraceptives other than condoms by WLHIV has also been documented in LAC and elsewhere 33, 34, 35. A possible explanation for the findings in some LAC countries could be that HIV care services are not integrated to SRHS due to the fragmentation in programmes and service delivery. This, limits the access to information and to contraceptive methods different from condoms, as so, WLHIV are not receiving adequate contraceptive counselling and the recurrent confusion between the concepts of “safe sex” and “contraceptive effectiveness” is persistent 13, 14, 21.

Studies conducted in Rwanda, Uganda and Swaziland have shown that contraceptive counselling offered by HIV services increases the use of dual methods, improves patient satisfaction, optimizes the work of providers and reduces healthcare costs 35, 37. The effects of adequate contraceptive counselling were also demonstrated by O'Reilly et al. where WLHIV who attended repeated contraceptive counselling sessions increased the use of dual methods. Consideration should be given to adapting these evidence‐based interventions to LAC countries with the aim of increasing service integration and improving the quality of care for WLHIV 33.

Sterilization is a controversial issue among WLHIV and has led to several forced sterilization cases taken to international courts. It is known that the immediate postpartum period is a vulnerable time for women, and particularly for those diagnosed with HIV during pregnancy or at delivery, limiting their possibility of making effective and objective decisions regarding future reproductive choices 33, 38. This review found that immediate postpartum sterilization remains higher for WLHIV in some LAC countries like Brazil, where c‐sections associated to HIV infection create an opportunity to have free access to this procedure as part of the obstetric care 9, 39. The review also showed that immediate postpartum sterilization is presented to WLHIV by prenatal care providers as a method to prevent perinatal transmission and as the best contraceptive alternative, which could be considered as an expression of poor quality SRH counselling, stigma and discrimination, and a form of institutional violence 10, 11, 14, 22.

Given the available interventions recommended by WHO, WLHIV can have planned pregnancies and vaginal deliveries with minimal possibility of perinatal transmission, and HIV seropositivity should never be a motivation for unwanted sterilization or abortion 5, 14. This review showed that WLHIV have higher rates of unplanned pregnancies besides high unsatisfied demand for contraception. Unplanned pregnancies resulting in induced abortion occurred twice as often in WLHIV compared to WNLHIV. In most Latin American countries, abortion is not only illegal but in some circumstances is considered an unacceptable practice, as motherhood holds very high religious, social, and cultural values 8, 14, 23. Although the reasons to terminate a pregnancy include well‐studied structural determinants, this review highlights that in LAC one of the main motives for WLHIV to undergo an induced abortion is the fear of perinatal transmission. This points again to the low quality of counselling during prenatal care and the great emphasis on screening pregnant women at the detriment to the screening of women of reproductive age when in contact with health services 8, 11, 40, 41.

This review also reports on structural barriers such as sexual and interfamily violence suffered by WLHIV when disclosing their serostatus to partners and family members, in addition to perceived stigma and discrimination that continues to permeate social and family relations in LAC 23, 42, 43. Similar results were described in South Africa where authors reported high levels of intimate partner violence in HIV‐serodiscordant couples 44 and by Kennedy et al., who demonstrated how interventions carried out with WLHIV, partners and families to facilitate disclosure of HIV status improve health outcomes, facilitate safe sexual behaviours and empower women to report violence. These highly effective interventions, adapted to their context, should be considered by LAC countries as part of the care provided in public health settings 30.

This review summarizes 18 studies with a total of 5672 WLHIV; nevertheless, it offers limited scope for generalization, and potential limitations to the review must be acknowledged. The studies included had mostly cross‐sectional observational designs, increasing the risk of selection bias 45. Also, since most of the studies were conducted in specialized HIV services, the results cannot be applied to the populations not using these services. Additionally, even though we applied the criteria of childbearing age (15 to 49) in the selection of studies, age subgroups were heterogeneously reported within the included studies, for this reason, we were able to make conclusions for the 25 to 35 range that represented the majority of the participants. The age group 15 to 24 was not assessed directly in most of the studies representing a limitation to our conclusions. Furthermore, 60% of the total study population originated from the city of Sao Paulo (Brazil), which could affect the generalization of the results, and only two studies are from Caribbean countries. Finally, difficulties in the completeness of data collection (national journals, grey literature) could have been a limitation.

Despite its limitations, this is, to our knowledge, the first systematic review of this subject performed exclusively with studies from LAC countries. Its value lies in the fact that its regional approach provides an overview of the local context, which could be used to design strategies or improve existing ones to expand access to and quality of SRH services by WLHIV.

The topics that emerged highlight the importance for LAC countries to apply the recommendations and good practice statements provided by the new guideline developed by WHO. These guidelines aim to create enabling environments for WLHIV with friendly health services that integrate SRH and HIV, granting sexual health counselling and support from trained and respectful healthcare providers and protecting women by developing violence prevention and safe abortion services 5.

## 5 Conclusions

WLHIV in LAC have unmet SRH needs related to structural barriers as well as limitations to access SRHS. It is paramount to address these restrictions from the perspective of the inalienable right to women's health and wellbeing, and the commitment to universal health coverage undertaken by the countries of LAC.

Improving the use of and access to SRHS among WLHIV dictates the need to provide safe preventive interventions during and prior to prenatal care, with modern contraceptive options and support for these women in making informed decisions.

This systematic review represents a contribution to existing knowledge since it is the first to exclusively focus on LAC, providing an overview of WLHIV sexual and reproductive needs information to support public health decision‐making.

## Competing interests

The authors state that they have no competing interest.

## Authors’ contributions

All authors contributed substantially to the conception, design, analysis and interpretation of data, and participated in the preparation and review of drafts of this manuscript, and gave their final consent to the publication of the final version.

## Supporting information


**Appendix S1.** Summary of studies included.
