# Supplementary material for: Access to sexual and reproductive health services for women living with HIV in Latin America and the Caribbean: systematic review of the literature
Source: J Int AIDS Soc. 2019 Apr 8;22(4):e25273. doi: 10.1002/jia2.25273 (PMC6452919; doi:10.1002/jia2.25273)
Supplement: Supplementary file 1 — Appendix S1. Summary of studies included. [file JIA2-22-e25273-s001.docx]

**Appendix 1.** **Summary of studies** **included**

**Quantitative studies**

| **ID** | **Country** | **OBJECTIVE** | **METHODS** | **ANALYSIS** | **RESULTS** |
| --- | --- | --- | --- | --- | --- |
| Aguliar et al, 2017  [25] | Mexico | Compare sociodemographic characteristics and the experiences of violence during pregnancy between WLHIV and WNLHIV. **Outcome**: unplanned pregnancies, sexual, physical and psychological violence. | Case-control study. Cases were WLHIV in prenatal care before or after delivery; controls were hospital-based WNLHIV attending two main referral public hospitals of Southern Mexico City; n= 152 women (50 WLHIV cases, and 102 WNLHIV controls). **Comparator**: HIV status. | Use of descriptive statistics (frequencies, medians, interquartile range). Univariate analysis and multivariate analysis using logistic regression to determine OR (adj.) [CI 95%]. | 38% of cases had their first pregnancy before the age of 18 vs 16% of controls OR=2.47 [1.07-5.72], also cases had more unplanned pregnancies than controls (20% vs. 16%), p=0.04. Last pregnancy was unwanted in 8% of cases compared to none in controls p=0.23. Cases reported higher levels of suffering any kind of violence including sexual, physical or psychological OR =16.05 [4.35-59.29. In multivariate analysis there was still strong association between HIV infection and age OR (adj.) =1.41 [1.17-1.72] and suffering violence OR (adj.) =11.18 [1.70-73.49]. |
| Barcellos et al, 2017  [10] | Brazil | Describe factors related to HIV post-diagnosis pregnancies. **Outcome**: occurrence of pregnancies after the diagnosis of HIV, unplanned pregnancies and experience of violence. | Cross-sectional study. Simple random sampling of WLHIV from all public specialized outpatient clinics in Porto Alegre; n=681 WLHIV. **Comparator**: women with and without pregnancies after the diagnosis of HIV. | Use of descriptive statistics; differences between two groups (Chi-square). Multivariate analysis with Poisson logistic regression to calculate Reasons of Prevalence (RP) [CI 95%]. | One third of WLHIV had at least one pregnancy after diagnosis. Post-diagnosis pregnancy was associated with a lower level of education RP (adj.) = 1.31 [1.03-1.66], being under the age of 20 RP (adj.) =3.48 [2.02-6.01], and having suffered HIV diagnosis-related violence RP (adj.) = 1.28 [1.06-1.56]. 65% of the post-diagnosis pregnancies were unplanned. |
| Barbosa et al, 2016  [8] | Brazil | compare the probability of obtaining female sterilization between WLHIV and WNLHIV (or unknown status) with at least one child. **Outcome**:  interval and postpartum sterilization; unmet demand for sterilization. | Secondary analysis from a cross-sectional quantitative study based on life course perspective (GENIH) study. The original study was conducted in the city of São Paulo, comparing two probabilistic samples of 975 WLHIV and 1,003 WNLHIV aged between 18 and 49 users of public health services.  **Comparator**: post-partum and interval sterilizations by HIV status. | Comparison of probabilities of sterilization using time-to-event analysis stratified by parity with multivariate-adjusted Cox model to calculate Hazard Ratios (HR) and OR [CI 95%]. | There was no significant difference in the risk of sterilization for women with at least one child between both groups HR=0.94 [0.69-1.29]. The risk for interval sterilization is 62% lower among WLHIV compared to WNLHIV HR=0.38 [0.18-0.80]. Postpartum sterilization in conjunction with c-section indicates a higher risk among WLHIV HR=1.94 [1.16-3.27]. 25% in both groups reported that they would like to have been sterilized previously, but unmet demand for sterilization is higher in WLHIV 30 years-old or younger OR (adj.) =3.08 [1.67-5.67]. |
| Kreitchman et al, 2015  [29] | Argentina, the Bahamas, Brazil, México, Perú | Describe multiparity among WLHIV and identify related factors. **Outcome**: repeat pregnancy. | Sequential multicentric cohort study.  WLHIV and their perinatally exposed HIV-uninfected infants prospectively followed in two sequential cohorts at 19 clinics in LAC that were part of the NISDI study; n=1,342 WLHIV. **Comparator**: occurrence of repeat pregnancy. | Descriptive statistics were used for demographic characteristics (median, interquartile range, frequency and percent). Multivariate logistic regression model analysis for associations measured with OR [CI 95%]. | Factors associated with increase occurrence of repeat pregnancy were: younger age, <26 years OR=1.07 [1.04-1.11] per one-year decrease in age); having a previous abortion OR=3.4 [1.4-8.4]. No association was found between repeat pregnancy and other factors such as the use of antiretrovirals, marital status, or education level. |
| Pilecco et al, 2014  [11] | Brazil | Compare WLHIV and WNLHIV in prevalence of induced abortion. **Outcome**: induced abortion, use of contraceptives, unplanned pregnancy. | Secondary data analysis of a cross-sectional study conducted in public health services in Porto Alegre with women aged 18-49. WLHIV were selected from all seven HIV specialized clinics; and WNLHIV from primary health services. Stratified sampling, n=1,123 women (625 WLHIV and 498 WNLHIV). **Comparator**: induced abortion by HIV status. | Use of descriptive statistics (percent, frequency) differences between groups using Chi-square. Binomial analysis with a GEE model to identify associations measured with OR [CI 95%]. | Difference in induced abortions among WLHIV vs. WNLHIV OR=2.92 [1.82-4.68]. More WLHIV used contraceptives OR=1.28 [1.11-1.40]; had more unplanned pregnancies OR=1.30 [1.02-1.66]; and more unplanned pregnancies ending in abortion OR=2.22 [1.50-3.29] when compared to WNLHIV. |
| Oliveira et al, 2014  [22] | Brazil | Determine the reasons given by WLHIV for being sterilized and identify related factors. **Outcome**: sterilization. | Cross-sectional study carried out in the HIV/AIDS outpatient clinic of the main public hospital in Fortaleza. All WLHIV aged 18-51 who attended the clinic in a three-month period were invited to participate; n=309 WLHIV. **Comparator**: pre- and post-diagnosis sterilization. | Comparison of medians using Mann-Whitney test, comparison of proportions using Fisher test. Multivariate logistic regression analysis for associations measured with OR [CI 95%]. | There were more postpartum sterilizations performed after the diagnosis at the c-section (96% vs 57%) OR=19.9 [5.51-72.8]. Interval sterilization was higher for women before diagnosis (43% vs 4%) OR=12.7 [4.19-38.9]. The main reason for sterilization before diagnosis was not wanting more children (52%) and after diagnosis was medical recommendation because of HIV (31.3%) and fear of perinatal HIV transmission (24%). 58% of the women had at least one child after diagnosis and were sterilized in the immediate postpartum. |
| Friedman et al, 2011  [12] | Brazil | Estimate predictors of pregnancy among WLHIV **Outcome:** pregnancy, induced abortions, violence. | Cross-sectional study nested in a cohort.  All WLHIV aged 18 or older receiving HIV care at the Instituto de Pesquisa Clínica Evandro Chagas (IPEC) were invited to participate; n=225 WLHIV. **Comparator**: occurrence of pregnancy. | Multivariate analysis logistic regression for predictors of pregnancy to determine OR [CI 95%]. | The pregnancy rate was 6.9% women/year (66% of the estimated national rate). Induced abortions were reported by 31 of the participants. 24% of women reported sexual abuse and domestic violence. The predictors of pregnancy were: 30 years or younger OR=3.42 [1.69-6.95], living with a partner OR=1.89 [1.0-3.57]. The higher the level of education lowered the risk of pregnancy OR=0.43 [0.19-0.99]. |
| Allen et al, 2010  [26] | Antigua and Barbuda, Barbados, Trinidad and Tobago | Identify factors associated with condom use. **Outcome**: condom use. | Cross-sectional study. All people living with HIV n=574 (260 women and 170 men but the analysis just takes the data from women) who attended health care centers in designated cities **Comparator**: condom use at last sexual intercourse. | Use of descriptive statistics (means, percentages), Multivariate analysis with logistic regression to determine OR [CI 95%]. | Data from women reported that use of condoms was associated with greater economic security OR=5.06 [1.47-17.39], having a serodiscordant partner OR=2.85 [1.28-6.33], and being married or having a permanent partner OR=2.86 [1.03-7.91]. |
| Barbosa et al, 2009  [9] | Brazil | Identify the characteristics of WLHIV and WNLHIV who have had induced abortions.  **Outcome**: prevalence of induced abortions, contraceptive use, sexual violence, sterilization. | Secondary analysis from a cross-sectional study in 13 municipalities on induced abortion with women aged 18 or older. WLHIV from specialized HIV clinics and WNLHIV from primary health care facilities were invited to participate; n=3,822 women (1,777 WLHIV and 2,045 WNLHIV) **Comparator:** HIV status. | Stratified uni and bivariate analysis. Adjusted logistic regression model to determine OR [CI 95%]. | More WLVIH had induced abortions (17% vs. 10%) p<0.0001 when compared to WNLHIV. The difference remained significant when age-adjusted, OR=1.96 CI 95% [1.60-2.39]. More WLHIV had sterilizations OR=1.46 CI 95% [1.24-1.32], used more male condoms OR=1.46 [1.26-1.69], less other contraceptive methods OR=0.26 [0.22-0.31]; suffered more sexual violence OR=1.47 [0.96-2.25] than WNLHIV. [Study conducted prior to PMTCT implementation in Brazil]. |
| Hopkins et al, 2005  [20] | Brazil | Compare WLHIV and WNLHIV regarding postpartum sterilization and incidence of medical recommendation to perform sterilization. **Outcome**: postpartum sterilizations. | Cross-sectional study in two cities with high HIV incidence rates (Porto Alegre and São Paulo). Stratified sample of two groups of women who attended prenatal care clinics; n= 825 (462 WLHIV and 363 WNLHIV). **Comparator:** postpartum HIV status. | Pearson’s Chi-square analysis was used to describe differences between groups. Multivariate analysis with logistic regression for predictors of sterilization, measured with OR [CI 95%]. | More WLHIV wanted to be sterilized than WNLHIV OR=4.70 [2.58-8.58]. The majority of WLHIV had sterilization during a c-section (68%) and had higher postpartum sterilization rates OR= 15.79 [8.77--28.4]. Sterilization occurred most often during c-section than vaginal deliveries OR=3.22 [1.69-6.13] among WLHIV when compared to WNLHIV. [Study conducted prior to PMTCT implementation in Brazil]. |

**Qualitative studies**

| **ID** | **Country** | **OBJECTIVE** | **METHODS** | **ANALYSIS** | **RESULTS** |
| --- | --- | --- | --- | --- | --- |
| Kendall, 2013  [14] | Mexico | Understand the contexts of unplanned pregnancies and access to contraceptive counseling for WLHIV. **Outcome**: characteristics of contraceptive counseling. | Qualitative interpretative study with discourse analysis based on grounded theory. WLHIV in outpatient clinics for management of HIV. Saturation sampling; n=55 WLHIV. | General sociodemographic questionnaire followed by in-depth interviews. Interpretive thematic discourse analysis. | 43/55 (78%) unplanned pregnancies. The only contraceptive offered was male condoms. No dual contraception alternatives offered and less than 2/55 (3.2%) use them. Pressure to undergo postpartum sterilization was persistent and based on medical recommendation as part of HIV prevention. No emergency contraception offered. |
| Van Dijk et al, 2014 [24] | Mexico | Explore the experiences of WLHIV regarding access to health services during pregnancy **Outcome**: barriers and facilitators to SRH. | Qualitative interpretive study with thematic discourse analysis. Theoretical saturation sampling of WLHIV attending hospitals and NGO clinics for people with HIV in four cities; n=32 WLHIV. | Analysis of demographic characteristics. In-depth interviews and focus groups. Interpretive thematic discourse analysis. | 20/32 (65%) of sterilizations in the immediate postpartum. 16/32 (50%) unplanned pregnancies. Also 16/32 (50%) of WLHIV with negative experience with health professionals due to pressure to undergo sterilization and 3/32 (10%) referred positive experiences but no counseling. |
| Chávez, 2015  [23] | Mexico | Explore sociocultural perceptions of WLHIV about unplanned pregnancy. **Outcome**: decision on continuing an unplanned pregnancy. | Qualitative interpretive study with discourse analysis. Snowball sample. Pregnant WLHIV attending the National Institute of Perinatology; n=15 WLHIV. | General sociodemographic questionnaire followed by in-depth interviews. Interpretive thematic discourse analysis using Atlas.ti. | 14/15 (90%) used condoms as only contraceptive. Little information on contraception and lack of knowledge about HIV led to inconsistent condom use. Most frequent reason for continuing pregnancy was the belief that abortion is a crime, additionally to social pressure. |
| Robicheaux et al, 2013 [27] | Dominican Republic | Understand the factors that influence the use of contraceptive methods by WLHIV to propose strategies that could reduce unplanned pregnancies. **Outcome**: unplanned pregnancies | Qualitative interpretive study. Convenience sample of WLHIV attending a maternity ward as part of the program for pregnant WLHIV or in the postpartum period; n=21 WLHIV. | General sociodemographic questionnaire. In-depth interviews and focus groups. Interpretive thematic discourse analysis. | For 13/21 (62%) of last pregnancies were unplanned and 17/21 (81%) of WLHIV did not want more children. The contraceptive methods used most often were pills (80.90%) and male condoms (66%). 20/21 (90%) had heard about other contraceptive methods. Social and religious influences reported as determining factor in the selection and use of contraceptives. The most important barrier was the perceived health effect of the contraceptive methods. |

**Mixed Studies**

| **ID** | **COUNTRY** | **OBJECTIVE** | **METHODS** | **SELECTION/ANALYSIS** | **RESULTS** |
| --- | --- | --- | --- | --- | --- |
| Gogna et al, 2009 [13] | Argentina | Describe the social situation of people living with HIV in Argentina. **Outcome:** contraception and reproduction needs. | Secondary analysis of data from the National study on the Social Situation of people living with HIV in Argentina. Mixed study: cross-sectional study followed by a qualitative interpretive study based on grounded theory. WLHIV for the cross-sectional were chosen after a multistage sample of public and private health services by region was performed n=296. Snowball technique with the collaboration of networks of people living with HIV was used for the qualitative stage n=23. | Quantitative use of descriptive statistics, frequencies expressed as percentage and standard deviations with CI 95%. Qualitative sequential thematic analysis of in-depth interviews and complementation methods. | 30% of WLHIV did not use condoms consistently due to rejection by partner. 79% used contraceptive methods (83% of them used condoms and 8% dual contraception).  55% of WLHIV did not want more children, and 52% of pregnancies that occurred after diagnosis were unplanned. 7% of WLHIV who wanted to have children did not discussed their intentions due to fear of disapproval of the healthcare provider. |
| Gogna et al, 2013 [15] | Argentina | Establish a baseline to identify obstacles in comprehensive treatment of WLHIV. **Outcome**: unwanted pregnancies, contraception, abortion. | Mixed study: cross-sectional and qualitative interpretive study. Convenience sample of WLHIV who had a consultation at a national referral center for HIV; n=369 WLHIV sampled and 11 took part in in-depth interviews. | Use of descriptive statistics with frequencies expressed as percentage and standard deviations with CI 95%. Sequential thematic analysis of in-depth interviews and complementation methods. | 40% of WLHIV became pregnant after diagnosis. 58% did not want more children. 50% had unplanned pregnancies. 63% use male condoms as the only contraceptive method. 60% of WLHIV reported difficulties in consistent condom use due limited ability to negotiate condom use with partners. 7% used dual contraception, with oral hormonal contraceptives used the most (6.3%). 55% had gynecological checkup in past year. 20% had received counseling on contraception. Emergency contraception was used at some point by 20% of WLHIV (80% of the interviewees new about it). |
| Montoya et al, 2014 [21] | Colombia | Analyze the performance of the PMTCT strategy and determine barriers to access health services. **Outcome**: quality of care, description of barriers to SRH. | Mixed study: cross-sectional and qualitative interpretive study. Convenience sample of pregnant WLHIV attending prenatal care; n=899 records of mother-child binomials exposed to HIV and 114 in-depth interviews. | Use of descriptive statistics (medians, percentages, and frequencies). Multivariate analysis with bivariate logistic regression to determine OR with 95% CI. Sequential thematic analysis. | 76% of WLHIV received antepartum ART, 86% had a c-section, 87% received intrapartum ART, 93% of infants received ARV prophylaxis, and 88% received formula for 6 months. 50% continued to receive ART for 13 weeks after delivery. The binomials that receive the five recommended interventions had 50 times less chance of perinatal HIV transmission. 30% did not receive counseling on contraception. After diagnosis, 80% had unplanned pregnancies. |
| Kendall et al, 2015 [28] | Honduras, Mexico, Nicaragua, Salvador | To analyze associations between social and economic characteristics and fertility history and experiencing pressure to sterilize from healthcare providers post-diagnosis among WLHIV. **Outcome**: postpartum sterilization. | Community-based mixed methods study with a convenience sample of 285 WLHIV from four Mesoamerican countries. WLHIV from professional and social networks, invited by leaders of the women’s health movement, (87 Honduras, 82 Mexico, 60 Nicaragua, 56 Salvador). Interviewer-applied questionnaire to elicit detail about WLHIV’s experiences with pressure by healthcare providers to accept a contraceptive method. | Thematic qualitative analysis of reports of how and when healthcare providers pressured women to undergo sterilization (from open-ended responses). Multivariate logistic regression to analyze the relationship between experiencing pressure to undergo sterilization and women’s social and economic characteristics and fertility history to determine OR [CI 95%]. | 23% of WLHIV experienced pressure to be sterilized post-diagnosis, ranging from 17% in Nicaragua to 28% in Mexico. WLHIV who had a pregnancy during which they and their healthcare provider knew their HIV status were more likely to experience coercive or forced sterilization than WLHIV who were not in these circumstances OR 5.66 [2.35-13.58]. The statistically significant predictors of being more likely to experience pressure to be sterilized were: having pregnancy with a known HIV-positive status and being in the youngest age group (<24 years-old). Qualitative analysis described WLHIV perceiving HIV status as a central motivation for healthcare providers to pressure them into sterilization to avoid HIV transmission, and presenting sterilization as a precondition for receiving medical services. |
